# Isolation and identification of excretory-secretory and somatic antigens from the *Oestrus ovis* larvae by SDS-PAGE and immunoblotting 

**Published:** 2014

**Authors:** Alireza Alborzi, Abbas Jolodar, Masoudreza Seyfi Abad Shapouri, Esmaeil Bagherian pour

**Affiliations:** 1 *Department of Pathobiology, School of Veterinary Medicine, Shahid Chamran University of Ahvaz, Ahvaz, Iran; *; 2 * Department of Basic Sciences, School of Veterinary Medicine, Shahid Chamran University of Ahvaz, Ahvaz, Iran.*

**Keywords:** Immunoblotting, *Oestrus ovis*, Sheep

## Abstract

*Oestrus ovis* is an economically important parasite of small ruminants and a zoonotic parasite with many reports of ophthalmomyiasis in human from Iran and other countries. The aim of the peresent study was the isolation and identification of excretory-secretory (ES) and somatic (S) antigens of *O. ovis* second and third stage larvae (L2, L3) collected from Arabi sheep breeds located in southwest of Iran. Positive sera were prepared by marking the sheep, taking blood sample and direct observation of the parasite in the head. Somatic antigens of the larvae (SL2, SL3) were prepared by sonication. Larval excretory-secretory antigens (ESL2, ESL3) were prepared by incubation the larvae in RPMI-1640 RPMI medium. Electrophoretic protein profiles of ESL2 two, ESL3 seven, SL2 eight, SL3 fifteen bands (from 79.0 to below 14.4 KDa) were shown. In immunoblotting with positive sera, four common bands in SL2 and SL3 at 58, 42.0, 29.0 and 28.0 kDa, one specific band in SL3 at 47.0 kDa and one band in ESL2, at 28.0 kDa**, **and three bands in ESL3 at 58.0, 42.0, 29.0 and 28.0 kDa were recognized. Among the profiles, the 28 kDa protein was the most common antigenic component. Nevertheless, the antigenic proteins 29, 58 kDa were a common protein in electrophoretic patterns of both S and ES proteins of L2 and L3 but, 42.0 kDa antigen the only one detected in immunoblot but not in S and ES protein profiles of the larvae. Therefore, the antigens 29.0, 42.0 and 58.0 kDa can be used for further studies of protective effects and serological diagnostic methods.

## Introduction

Infection or infestation of the body hosts (human or animals) by the larval stages of dipterous flies – myiases – is of medical and veterinary importance.^1^ *Oestrus ovis *(Linné 1761) is the most well-known and economically important species of genus*, Oestrus* in nine genera of the subfamily Oestrinae.* Oestrus ovis* larvae are obligatory parasites and cause nasal myiases of sheep and goats**. **It is also a zoonotic parasite because the larvae may cause human pharyngeal myiasis or external ophthalmomyiasis as many cases reported from Iran and other countries.^1^^-^^8^ The parasite is now found in all sheep-farming areas of the world. The larvae in infected sheep can cause histhopatological changes in nasal tissues, allergic and inflammatory responses, followed by bacterial infection and sometimes death. When the adult females attack the sheep to deposit larvae, the activity of them, may annoy the animals, leading to feeding stop, reduced weight gain in lambs and loss of condition. Infection by the parasite has been associated with losses in weight gain (1 to 4.5 kg), losses in wool production of up to 200 to 500 g and a reduction in milk production of up to 10.0%, since the parasitic larvae also seriously impact the well-being and productive performance of their hosts causing loss in the production.^1^^,^^2^^,^^9^ Prevalence of infection tends to be highly localized. In individual sheep flocks infection rates of up to 44.0 to 88.0% have been recorded in France and as low as 0.7% in Britain. Infection rates of 6 to 52.0% have been recorded in Zimbabwe, 69.0% in India and 100% in Morocco, South Africa, and Brazil, 40.3% in Turkey, 49.7% in Iran, 17.2% in Iraq.^1^^,^^2^^,^^10^^-^^12^ The ES products of the myiasis- producing larvae are usually involved in nutrition and their development. These products are often immunogens.^13^ Immunological studies on the diagnosis of Oestrus* ovis* infection in sheep has been carried out using larval extracts as antigen sources by indirect hemagglutination,^14^ enzyme-linked immuno-sorbent assay (ELISA).^15^^-^^18^ Although, Tabouret *et al.* indicated that a 28.0 kDa protein complex (pc28) was the main antigenic component of ES products from larvae (L2 and L3).^19^ Moţ in study of the antigenic structure characterization of *O. ovis* larvae (L2 and L3) emphasized six antigenic fractions with molecular weights situated in 27.0 to 80.0 kDa domain, with two clear and intense fractions.^20^ Therefore, the aim of this study was the isolation and identification of ES and somatic (S) antigens of *O. ovis *larvae (L2 and L3) collected from Arabi sheep breeds of southwest of Iran to find similarities or possible differences among antigens based on host and the parasite of the area.

## Materials and Methods

**Animals and sera. **Sera and *O. ovis* larvae were prepared from sheep in the slaughterhouse of Ahvaz, Khuzestan province, Iran. The sheep population in Khuzestan province is around 4 million heads. About 30.0% of them were Arabi breed.^21^ Blood samples were taken individually form the animals and transferred to the parasitology laboratory of the veterinary faculty. The heads of slaughtered sheep were examined for *O. ovis* larvae by cutting the horn and hitting the heads on the ground several times. The collected larvae were identified according to entomological keys described by Zumpt,^22^ then sera of each infected heads marked as positive sheep sera and stored at – 20 ˚C until use. Negative sera were prepared from indoor lambs (up to 3 months of age). 

**Preparation of antigens. **The second and third stage larvae (L2, L3) of the parasite were collected and washed several times in phosphate-buffered saline (PBS) supplemented with antibiotics (100 U mL^-1^ of penicillin G potassium and 100 mg mL^-1 ^of streptomycin). The viability of larvae was checked under a stereomicroscope. The somatic crude extracts (S) were prepared as follows: A total number of 10 second (L2) and one third (L3) stage larvae were separately sonicated with a Bandelin sonicator (Bandelin, Berlin, Germany) in 5 mL PBS pH 7.2, with 5 mM DTT (Dithiothreitol, Thermo Fisher Scientific, Waltham, USA) and then centrifuged at 2000 *g* for 15 min at 4 ˚C. The extract was filtered through 0.22 mm filters (BIOFIL Syringe Filter, Shanghai China) and finally stored at –20 ˚C until use. The excretory-secretory products (ESP) were obtained from the culture of the two different larval stages *in vitro*. Batches of five live L2 were maintained in a 75 cm^2^ cell culture flask (Greiner Bio-One, Solingen, Germany), containing 10 mL Roswell Park Memorial Institute medium (RPMI-1640; Bahar Afshan, Tehran, Iran) with penicillin G potassium and streptomycin. In the case of L3, five L3 were maintained in the flask containing 20 mL of the culture medium. Larvae were incubated in darkness for 24 hr in 5.0% CO_2_ at 37 ˚C. Supernatants were collected, centrifuged at 2000 *g* for 15 min at 4 ˚C, filtered through 0.22 mm filters and stored at –20 ˚C until use.

**Electrophoretic analysis. **Samples of the somatic crude extracts and ESP of second and third stage larvae (named SL2, SL3 and ESL2, ESL3 respectively) were separated by sodium-dodecyl-sulfate- polyacrylamide gel electrophoresis (SDS-PAGE) based on Laemmeli's method in an 8.0% separating gel using electrophoresis apparatus (Paya Pajoohesh Pars, Tehran, Iran).^23^ Prior to electrophoresis, the samples were mixed with an equal volume of a non-reducing sample buffer and boiled for 5 min then centrifuged for 2 min. Unstained molecular mass standards (Pierce) ranging from 116 to 14.4 kDa were used. Electrophoresis was run at 100 V for 5 hr under reducing conditions. SDS-polyacrylamide gel was stained for protein visualization with Coomassie blue.

**Immunoblotting. **For immunoblotting, proteins of SL2, SL3 and ESL2, ESL3 were first electrophoresed on 8.0% SDS-polyacrylamide gel. Western blotting was carried out following established protocols.^24^ Proteins were transferred onto a nitrocellulose membrane using tris-glycine-methanol buffer for 1.5 hr at 12 mA. The membrane was blocked in 5.0% skim milk in PBS for 1h at room temperature. After rinsing three times in the washing buffer (PBS-T: PBS, 0.05% Tween 20) the membranes were washed once in PBS. Sera (5 positive and 2 negative) were diluted (1:50, 1:100, 1:200, 1:400 and 1:1000) in PBS-T buffer and the membranes incubated with stirring at room temperature (RT) for 1 hr and again washed three times with PBS-T for 10 min each time. Secondary antibody peroxidase labeled (i.e. anti-sheep IgG; Sigma-Aldrich, St. Louis, USA) was diluted in PBS-T (1:1000 - 1:5000) and incubated for 1 hr at RT and developed in a mixture of 4-cloronapthol and hydrogen peroxide. 

## Results

**Fig. 1:**
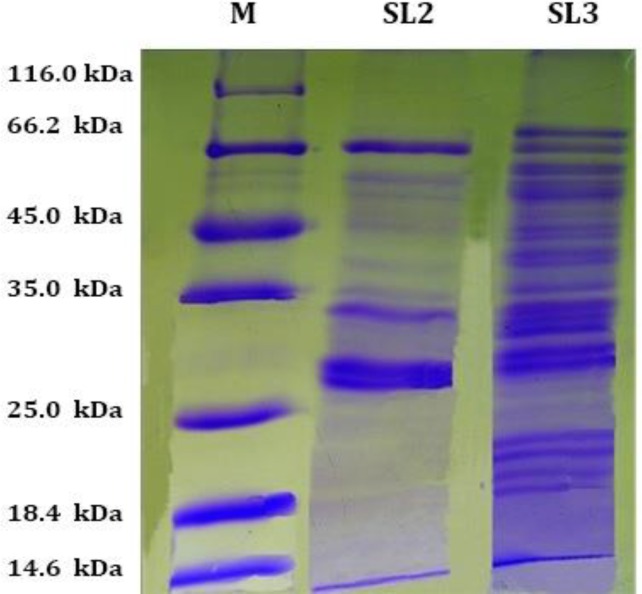
SDS-PAGE in reducing conditions. Acrylamide 8.0%. Somatic antigens of second (SL2), and third larvae (SL3) of *Oestrus ovis*, eight bands from 66.0, to a band below 14.6 kDa at SL2 lane. More than fifteen bands from 79.0 to a band below 14.6 kDa at SL3 lane. Molecular weight marker (M).

**Fig. 2:**
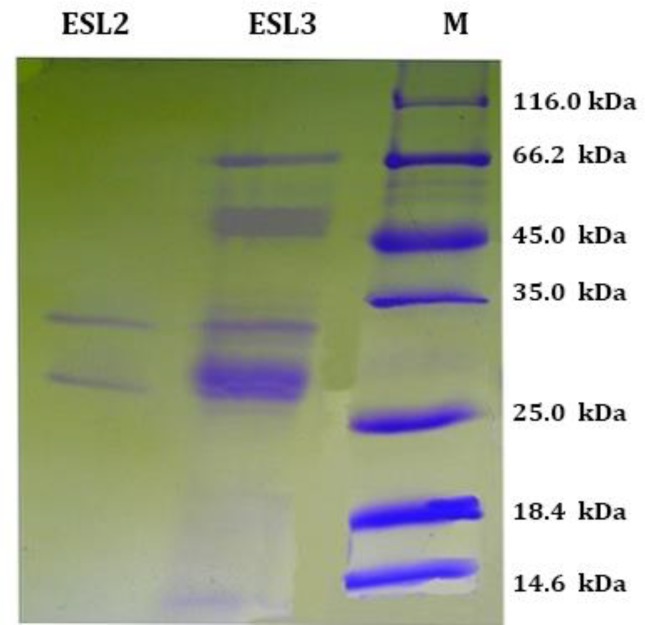
SDS-PAGE in reducing conditions acrylamide 8.0%, excretory-secretory antigens of second (ESL2), and third larvae (ESL3) of *Oestrus ovis*, two bands in ESL2 lane at 32.0, 28.0, seven bands in ESL3 lane from 66.0 to below 14.6 kDa molecular weight marker (M).

**Protein profiles of S and ESP. **SDS-PAGE analysis of somatic extracts and ESP of *O. ovis* larvae (L2 and L3) revealed the presence of a complex array of protein bands ranging in molecular weight from 79.0 to a small peptide below 14.6 kDa. The predominant S protein bands were eight bands at 66.0, 58.0, 39.0, 35.0, 34.0, 28.0, 27.0 and a band below 14.6 kDa of SL2; more than fifteen bands at 79.0, 66.0, 58.0, 45.0, 42.0, 39.0, 34.0, 28, 27.0, 23.0, 22.0, 21.0, 19.0, 18.0 and a band below 14.6 kDa of SL3 (Fig. 1). The predominant ES protein bands were two bands at 32 and 28.0 of ESL2 and seven bands at 66.0, 58.0, 47.0, 32.0, 28.0, 26.0, below 14.6 kDa of ESL3 (Fig. 2).The electrophoretic patterns of the larval S and ES proteins (L2 and L3) showed a marked homologous band at 28.0 kDa, particularly present in SL2, SL3 and ESL3 to a lesser extent in ESL2 (Figs. 1 and 2).

**Fig. 3:**
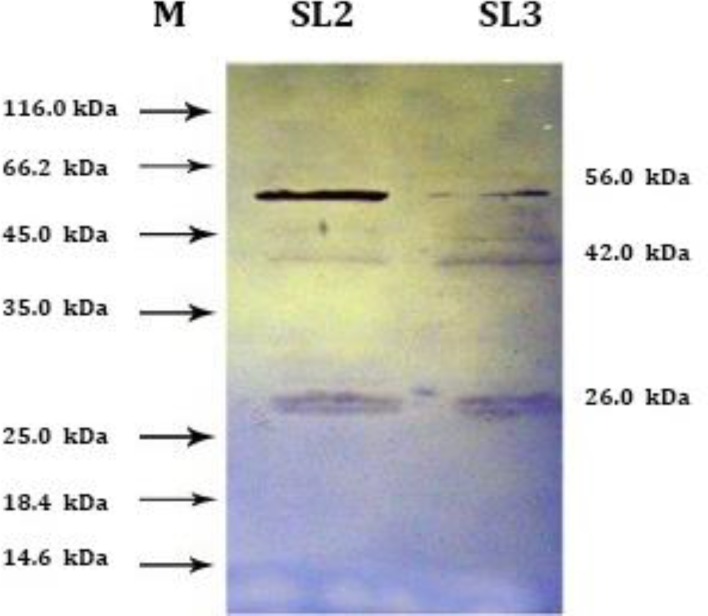
Immunoblotting of somatic antigens from *Oestrus ovis* second (SL2), and third stage larvae (SL3) with serum of a naturally infected sheep. Four common bands in SL2 and SL3 lanes at 58.0, 42.0, and a double band 29.0, 28.0 kDa. One specific band in SL3 at 47.0 kDa. Molecular weight marker (M).

**Fig. 4:**
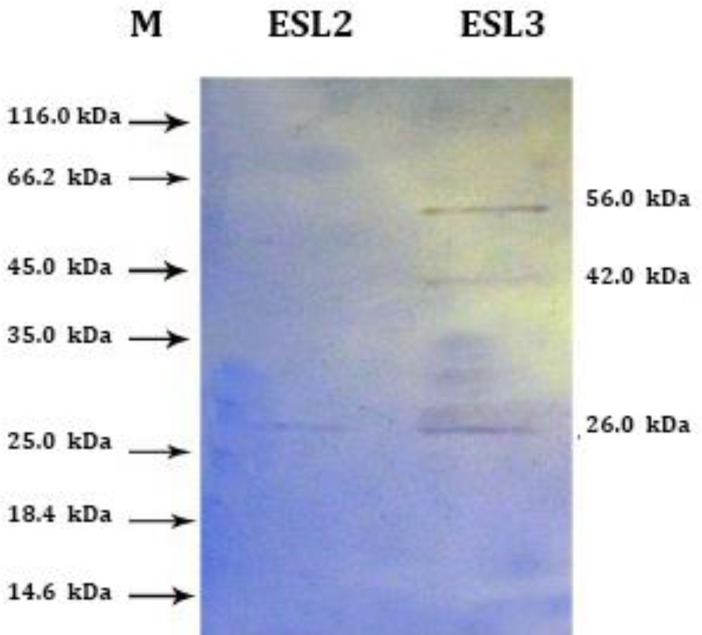
Immunoblotting of excretory-secretory antigens from *Oestrus ovis* second (ESL2), and third stage larvae (ESL3) with serum of a naturally infected sheep. One band in ESL2 lane, at 28.0 kDa, and three bands in ESL3 lane at 58.0, 42.0, 29.0 and 28.0 kDa (two latter as a double band) Molecular weight marker (M).

**Immunoblotting. **Analysis of electrophoretic patterns in immunoblotting of somatic extracts and ESP of *O. ovis* larvae (L2 and L3) with the 1:50 dilution of naturally infected sera and 1:1000 dilution of anti-sheep IgG conjugate, positive reactions (as visible bands) were obtained. The results demonstrated that the predominant S antigenic bands were four common bands in SL2 and SL3 at 58.0, 42.0, 29.0 and 28.0 kDa (two latter as a double band). One specific band in SL3 at 47.0 kDa with strongest reaction at 28.0, 29.0 and 42.0 kDa (Fig. 3). However, the predominant ES antigenic bands were one band in ESL2, at 28.0 kDa, and three bands in ESL3 at 58.0, 42.0, 29.0 and 28.0 kDa (two latter as a double band) with strongest reaction at 28.0, 42.0 and 58.0 kDa (Fig. 4). The result showed that some protein of S and ES were antigenic because they were recognized by naturally infected sheep. Among the protein profiles, the 28.0 kDa protein was the most common antigenic component.

Nevertheless, the 29.0 and 58.0 kDa antigenic proteins were a common protein in electrophoretic patterns of both S and ES proteins of L2 and L3 but, 42.0 kDa antigen only detected in immunoblot, not seen in S and ES protein profiles of the larvae. Immunoblotting analysis gave negative reactions with sera from indoor lambs.

## Discussion

In the present study, we used ES and somatic proteins of the second and third-stage larvae as the antigenic source, since they were present in the sheep for a long time, they were easily collected and they contained a large amount of protein. We observed five immunogenic proteins in larval somatic crude extracts and ESP of* Oestrus ovis* with using sera of naturally infected Arabi sheep from southwest of Iran and anti-sheep IgG conjugate. This showed when the infection occurred. The immunogenic proteins stimulate the production of humoral antibodies especially systemic IgG. Several studies have been done on immunoresopnse of sheep to *O. ovis* infection, antigenicity of larval crude extracts and ESP of the parasite and use of them for serological diagnostic purposes.^25^^,^^26^ Frugere *et al.* reported that ESP of L3 and crude extract of L2 are antigenic with different optical densities of an antigen of them.

These findings suggest that specific antibodies could be induced and could interfere with the extracorporeal digestion and consequently with larval growth. ^13^ Suarez *et al*. analyzed antibody responses (IgG and etc) against *O. ovis* in sheep and in lambs from Sardinia (Italy) by an indirect-enzyme-linked immunoassay test and L2 *O. ovis* ES antigens. They observed a positive significant correlation in sheep between *O. ovis* L2 and the IgG optical densities.^27^

Several electrophoretic analysis of proteins of *O. ovis* larvae (L2 and L3) and immunoblot studies have been conducted with some similarities and some differences in results of the protein profiles and antigenic activity of them in immunoblotting tests. Innocenti *et al*. by gel electrophoresis, indicated that larval cuticular extract of *O. ovis* 3^rd^ instar larvae (L3) contain a number of polypeptides ranging in molecular weight from 180.0 to 4.5 kDa. In immunoblotting test, the cuticular polypeptides most reactivated against sheep antisera were in the range of 180.0 to 56.0 kDa.^28^ In a study by Taburate *et al*. electrophoretic analysis of proteins from four different extracts of *O. ovis* L2 in 15.0% acrylamide SDS-PAGE under reducing conditions revealed homologous bands between the extracts.^19^ The crude extract (L2CE), presented a complex array of bands ranging from 84.0 kDa to small peptides below 14.0 kDa. The electrophoretic patterns showed a marked homologous band at 28.0 kDa, particularly present in ESP, and salivary gland contents (SGc) and to a lesser extent in digestive tube contents (DTc) and L2CE. Moreover, a 39.0 kDa homologous band was also detected in SGc, ESP and L2CE, but not in DTc. In immunoblotting test of the patterns with experimentally infected lambs demonstrated that SGc and ESP were highly antigenic. In contrast, DTc and L2CE were less recognized by infected animals. The 28.0 kDa protein was the most antigenic component of all extracts. An 84.0 kDa antigen was also weakly detected for almost all sera.^19^ Angulo-Valadez *et al*., by electrophoresis under non-reducing conditions, showed that the protein pattern of salivary gland products of *O ovis* L3 include several bands between 26.0 and 84.0 kDa, with major bands at 28.0, 29.0, and 84.0 kDa. The most antigenic activity occurred at the 29.0 kDa band.^25^ Moţ, through SDS-PAGE electrophoresis (undefined method conditions), indicated that *O. ovis* larvae (L2 and L3) contain six domain fractions with molecular weights situated in 27.0 to 80.0 kDa.^20^ In present study, in 8.0% acrylamide, under reducing conditions, electrophoretic analysis of S proteins and ESP of *O. ovis* larvae (L2 and L3) revealed the presence of a complex array of protein bands ranging in molecular weight from 79.0 to a small peptide below 14.6 kDa. 

In immunoblotting with naturally infected Arabi sheep sera five bands were recognized includeing 58.0, 47.0, 42.0, 29.0 and 28.0 kDa in proteins of *O. *ovis larvae (L2 and L3) collected from the animals. Among the protein profiles, the 28.0 kDa protein was commonest antigenic component. The results are almost similar with the findings of Taburate *et al*. and Angulo-Valadez et al.^19^^,^^25^ Nevertheless, the antigenic proteins 29.0, 58.0 kDa were common proteins in electrophoretic patterns of both S and ES proteins of L2 and L3 but, 42.0 kDa antigen was the only one detected in immunoblot, not seen in S and ES protein profiles of the larvae. As a general rule, differences in the number and range of molecular weight proteins in electrophoretic patterns and consequently their immunoblotting patterns may be caused by variations in extraction method, used media and analysis conditions. Therefore, despite use of a purified 28 kDa protein complex (pc28) for diagnostic purpose in a direct ELISA test,^19^ the present study indicated that other proteins (29.0, 42.0 and 58.0 kDa) could also be considered in serological diagnostic and immuno-protective studies on* O. ovis* infection in sheep.
